# Social determinants of hazardous alcohol use in a Ugandan population cohort

**DOI:** 10.1080/16549716.2025.2484870

**Published:** 2025-04-10

**Authors:** Leo Ziegel, Carl Fredrik Sjöland, Erinah Nabunya, Robert Bulamba, Emmanuel Kyasanku, Stephen Mugamba, Godfrey Kigozi, Alex Daama, Grace Kigozi, Amanda P. Miller, Anna-Clara Hollander, Anders Hammarberg, Fred Nalugoda, Anna Mia Ekström

**Affiliations:** a Department of Global Public Health, Karolinska Institutet, Solna, Sweden; b Public Health Agency of Sweden, Solna, Sweden; c Africa Medical and Behavioural Sciences Organization, Nansana, Uganda; d School of Public Health, San Diego State University, San Diego, CA, USA; e Centre for Psychiatry Research, Department of Clinical Neuroscience, Karolinska Institutet and Stockholm Health Care Services, Region Stockholm, Stockholm, Sweden; f Department of Infectious Diseases (Venhälsan), South General Hospital, Stockholm, Sweden

**Keywords:** Alcohol consumption, alcohol use prevalence, social factors, socioeconomic factors, urbanity, Low- and middle-income country, Sub-Saharan Africa

## Abstract

**Background:**

There is a limited population-based data on hazardous alcohol use and associated social determinants in many African countries.

**Objectives:**

To examine patterns of hazardous alcohol use across a range of social determinants of health in Uganda, with a particular focus on gender differences.

**Methods:**

This cross-sectional study used data collected in 2021–2022 from an open population cohort spanning urban, semi-urban, and rural communities. Alcohol use was assessed with the Alcohol Use Disorders Identification Test-Consumption (AUDIT-C). Covariates were selected according to the WHO’s Social Determinants of Health framework. Poisson regression with robust variance was used for data analysis.

**Results:**

Of the 3459 participants, 2085 (60%) were women. Overall, the prevalence of hazardous alcohol use was 5% among women and 18% among men. Strong positive associations with hazardous use were found for individuals residing in semi-urban areas (female aPR 2.1 [95% CI 1.3–3.3], male aPR 1.8 [95% CI 1.4–2.5]), past-year perpetration of intimate partner violence (female aPR 2.2 [95% CI 0.8–5.6], male aPR 1.4 [95% CI 0.9–2.2]), smoking, middle age for men (aPR 1.6 [95% CI 1.2–2.2]), and employment as a vendor in a restaurant or bar for women (aPR 1.5 [95% CI 1.0–2.2]). Strong negative associations were found for high educational attainment, Muslim or Pentecostal religion, and living in a marriage or union for women (aPR 0.7 [95% CI 0.5–1.0]).

**Conclusions:**

Hazardous alcohol use was prevalent, especially among men, in a representative Ugandan population sample. Specific target groups for public health and clinical interventions were identified, such as women working in the hospitality sector. Residents of semi-urban communities may encounter unique risks for hazardous alcohol use, compared with rural and highly urban populations.

## PAPER CONTEXT

- **Main findings**: Hazardous alcohol use is prevalent in a representative Ugandan population, particularly in semi-urban communities.
- **Added knowledge**: These findings contribute to the growing evidence on the role of social determinants in shaping alcohol use in low- and lower middle-income countries.
- **Global health impact for policy and action**: Policy makers should prioritize addressing social determinants to mitigate the substantial burden of hazardous alcohol use.

## Background

Alcohol use is a leading global risk factor for negative health outcomes and social harms [1,2], causing worldwide economic losses on a scale of hundreds of billions of USD annually [3]. Reducing harmful alcohol use is included in the Sustainable Development Goals as a key strategy to improve the overall global well-being [4], and the WHO recommends that countries offer evidence-based interventions to prevent the progression from hazardous to harmful use (see Box 1 for definitions) [5].

**Box 1.** Definitions of terms [5,7].

*ICD-11 = International Classification of Diseases 11th Revision, DSM-5 = Diagnostic and Statistical Manual of Mental Disorders, Fifth Edition.*
*Hazardous Alcohol Use*: Health risk factor according to ICD-11, describing consumption patterns that substantially increase the risk of negative physical or mental health consequences for the user or others. *Harmful Alcohol Use*: ICD-11 diagnosis for consumption that has resulted in adverse physical or mental health consequences for the user or others. Used in public-health contexts, harmful use also encompasses negative social consequences. *Alcohol use disorders*: ICD-11 diagnostic group for Harmful Alcohol Use and Alcohol Dependence. Both disorders are covered by the DSM-5 diagnosis of Alcohol Use Disorder. Alcohol Dependence and Alcohol Use Disorder encompass both health and social factors. The abbreviation ‘AUD’ is used for alcohol use disorder(s) as defined in both ICD and DSM.

Alcohol exerts its harmful effects through pathways shaped by social determinants of health – factors fundamental to people‘s daily lives, such as education, occupation, and social cohesion [6]. However, there is limited population-based data on the associations between social determinants of health and alcohol use from low- and lower middle-income countries, including most African nations [8–10]. Most existing evidence derives from high- or upper middle-income settings, where higher socioeconomic groups typically consume more alcohol but experience fewer alcohol-related harms than lower socioeconomic groups [9,11–14]. In African countries, these patterns are less clear due to the scarcity of large-scale population studies [10,15]. Moreover, discrepancies in reported data and the use of heterogeneous measures (see Box 1) complicate accurate assessments of alcohol-related harms.

A 2023 review identified being male, having a low level of education, and earning a low income as key social determinants linked to alcohol use disorder (AUD) in sub-Saharan Africa [16]. However, the generalizability of these findings was limited, as most studies included in the review were not population-based. A 2024 meta-analysis focusing on East Africa estimated a pooled AUD prevalence of 16%, with men experiencing AUD 2.7 times more than women [8,17]. Yet, the authors advised caution in generalizing patterns of social determinants and AUD across a region undergoing substantial population growth, economic development, cultural shifts, and climate change.

According to the WHO, harmful alcohol use is increasing in African countries [18,19], and among them Uganda has the highest estimated per capita consumption on the continent [5]. The 2014 STEPS survey, which is the largest nationally representative study on alcohol use to date, found an AUD prevalence of 9.8%. Being male, older age, and lower educational attainment were the social determinants that are most strongly associated with AUD, with evidence of substantial geographic heterogeneity in these associations [20]. In the most recent national STEPS survey (2023), 13% of men and 7% of women reported daily alcohol consumption during the past year [21]. Smaller studies in rural Uganda have identified additional factors associated with AUD, such as being Christian (in contrast to the country‘s significant Muslim population), currently being or having been married, working as a fisherman, or as a woman employed in bars or restaurants. Nuancing international findings, local results also indicate that a higher socioeconomic status is associated with AUD [22,23]. Despite considerable progress in combatting HIV, Uganda has been and remains highly affected by the generalized HIV epidemic in East Africa. Alcohol consumption is a well-established risk factor for both acquiring HIV through unsafe sexual practices and for poor treatment adherence among those living with HIV [24].

Given the paucity of high-quality population-based data on hazardous alcohol use from many African countries and contradictory findings regarding the role of social determinants, this study aimed to explore patterns of hazardous alcohol use across a range of social determinants of health in a Ugandan population cohort, with a particular focus on gender differences.

## Methods

Data were collected from May 2021 to July 2022 as a part of the third round (R3) of the Africa Medical and Behavioural Sciences Organization (AMBSO) Population Health Surveillance (APHS). APHS is an open population cohort with a yearly household census and individual surveys, covering multiple health and behavioural outcomes. A detailed cohort description has been published previously [25]. APHS received ethical approval from local Clarke International University (CIUREC/0059) and is registered with the Ugandan National Council for Science and Technology (UNCST/SS4468).

### Setting

APHS includes six distinct communities, including one rural, one semi-urban, and one urban site in each of the Ugandan districts of Hoima and Wakiso. Hoima is situated in Western Uganda, and Wakiso encircles the capital city, Kampala, in central Uganda. Study communities were selected to represent the diversity of Ugandan society, including interregional differences, according to variation in demographics, public and commercial service availability, and distribution of income-generating activities. The definitions of community types are further elaborated in the cohort description [25]. Urban communities are defined as those located near or inside urban centres, while in rural communities, households are typically separated by at least 100 m. The definition of semi-urban communities aligns closely with that employed by UN-Habitat for peri-urban communities: ‘belonging to urban clusters but not part of a town’ [26]. APHS is the only Ugandan population cohort covering urban, semi-urban, and rural sites.

### Data collection

First, all households in the study communities were mapped with inhabitants censused at >99% coverage. Second, all eligible censused inhabitants who were at least 13 years of age were invited through community outreach meetings and recurrent community visits to a nearby data collection hub for the individual survey. Participants were offered general health screenings as well as blood sampling (including HIV testing), after which same-sex research assistants administered face-to-face questionnaires in Luganda language (in Wakiso) or Runyoro language (in Hoima), or in English, as per the preferences of each participant. The population cohort included 18,584 censused individuals aged ≥18 years, of which 3575 participated in the survey, yielding a 19% participation rate. After data cleaning, 116 participants (3%) had incomplete records and were excluded, giving a final sample of 3459 individuals.

### Measurements

In this study, the main outcome was hazardous alcohol use measured with the Alcohol Use Disorders Identification Test-Consumption (AUDIT-C) [27]. AUDIT is a WHO-developed questionnaire used to screen for hazardous alcohol use and other alcohol-related conditions using varying cutoffs. AUDIT has been validated as a severity indicator of alcohol dependence in Uganda [28]. AUDIT-C is a short-form AUDIT, comprised AUDIT questions 1–3 on consumption measured in standard drinks, and is highly correlated to the full AUDIT score [29]. All APHS participants were asked to describe their consumption over the previous 12-month period, and this information was translated into standard drinks by research assistants using a card depicting the most common alcoholic beverages in Uganda.

There are no universally agreed cutoff points for AUDIT-C. Based on previous research to balance sensitivity and specificity [30], hazardous alcohol use was defined as scoring ≥3 points for females and ≥4 points for males. Descriptive results were also calculated for a higher cutoff of ≥4 points for females and ≥5 points for males, indicating more harmful alcohol use.

Covariates were selected in accordance with the WHO’s social determinants of health framework to capture both more distal (or structural) and more proximal (or intermediary) factors [6]. Additionally, lab-verified HIV status was included.

#### Distal covariates

*Gender* was determined by interviewers as man or woman, given the sensitive cultural and legal context. *Age* was self-reported in completed years and categorized into 18–20, 21–29, 30–39, 40–49, 50–59, and 60+ age groups. *Highest education* level attended was categorized into none, lower primary (P1–P4), upper primary (P5–P7), lower secondary (S1–S4), upper secondary (S5–S6), and tertiary. *Occupation* was reported as work or daily activity, if several activities were reported the first one mentioned was selected. Activities were categorized according to International Labour Organization (ILO) and Demographic and Health Surveys program (DHS) methodologies, into professional employment, sales and services, skilled manual work, unskilled manual work, agriculture, transport, and other [31,32]. *Religion* was reported as none, Catholic, Protestant, Saved/Born again or Pentecostal, Muslim, or other. None and Saved/Born again or Pentecostal were grouped in ‘other’ in the analysis.

#### Proximal covariates

For m*arriage or union*, participants were asked if they currently live in a marriage or union, and whether this was a traditional, religious, consensual or civil marriage, or union. Consensual unions include cohabiting. *Family size* was the reported total number of living children. Household *socioeconomic status* was assessed using asset indices which were calculated according to standard methodology from the DHS, both stratified by urbanity status and for the entire sample [33]. *Urbanity* status of study communities was defined at cohort creation according to *inter alia* density of households, the existence of shops, places for social gatherings, employment opportunities, and means of transportation. *Smoking* was asked as current or ever use of cigarettes, pipes, or other tobacco. To assess *intimate partner violenc*e (IPV), participants were asked whether they had committed emotional, physical, or sexual violence against their sexual partner (perpetration), and whether they had experienced such violence by their sexual partner (victimization), in the past 12 months, using prompted standard response options developed by the WHO [34].

#### HIV status

*HIV serostatus* was determined using the Uganda Ministry of Health testing algorithm, which includes a Determine HIV-1/2 Strip (Abbott Laboratories), followed by a confirmatory test using an HIV 1/2 STAT-PAK Assay (Chembio Laboratories) for positive cases, and an SD Bioline HIV-1/2 3.0 test (Abbott Laboratories) if the results from the first two tests are inconsistent [35].

### Statistical analyses

The authors followed a complete case analysis approach. No *a priori* hypotheses were formulated, but a gender-stratified analysis was planned *a priori* due to the expectation of hazardous alcohol use and its determinants being highly gendered. This expectation was confirmed by highly statistically significant differences between women and men in total AUDIT-C score means (using Student‘s t-test) and binary categorizations (using Pearson‘s chi-squared test and Fisher‘s exact test) (*p* < 0.001).

First, descriptive statistics were calculated for all covariates, and *p*-values were assessed for the null hypothesis of no difference in AUDIT-C status between covariate levels using Pearson‘s chi-squared test and Fisher‘s exact test. These tests yielded nearly identical results, with the former‘s results reported below. Bivariate tabulations and crude analyses identified small cell numbers; thus, several covariate levels were collapsed to improve multivariable model stability.

Associations between hazardous alcohol use and covariates were estimated using Poisson regression with robust variance estimation [36,37], yielding prevalence ratios (PRs) with 95% confidence intervals (CIs).

Crude (unadjusted) regression models were calculated separately for all gender-covariate combinations. Thereafter, covariates were sequentially combined in a step-up approach, assessing goodness-of-fit with Akaike information criteria and Bayesian information criterion. Potential interactions between covariates were assessed without substantially improving model fit.

Analyses were performed using Stata 18.0 SE and R 4.4.1.

## Results

Descriptive statistics of the 3459 participants is shown in Table 1.

**Table 1.** Descriptive statistics of the study sample (*n* = 3459), by gender and hazardous alcohol use.

	WOMEN			MEN			TOTAL		
VARIABLES	AUDIT-C ≥ 3 (*n* = 114)	Total (*n* = 2085)	p-value	AUDIT-C ≥ 4 (*n* = 247)	Total (*n* = 1374)	p-value	AUDIT-C ≥ 3 F/≥4 M (*n* = 361)	Total (*n* = 3459)	p-value
**Age**			0.062			<0.001			<0.001
18–20	9 (3.7%)	242 (11.6%)		7 (4.7%)	148 (10.8%)		16 (4.1%)	390 (11.3%)	
21–29	39 (5.5%)	712 (34.1%)		70 (14.5%)	484 (35.2%)		109 (9.1%)	1196 (34.6%)	
30–39	36 (6.8%)	529 (25.4%)		74 (22.0%)	337 (24.5%)		110 (12.7%)	866 (25.0%)	
40–49	20 (7.0%)	285 (13.7%)		48 (23.2%)	207 (15.1%)		68 (13.8%)	492 (14.2%)	
50–59	9 (4.9%)	182 (8.7%)		36 (31.9%)	113 (8.2%)		45 (15.3%)	295 (8.5%)	
60+	1 (0.7%)	135 (6.5%)		12 (14.1%)	85 (6.2%)		13 (5.9%)	220 (6.4%)	
**Highest education**			0.009			<0.001			<0.001
None	7 (8.3%)	84 (4.0%)		18 (24.3%)	74 (5.4%)		25 (15.8%)	158 (4.6%)	
Lower primary	18 (7.6%)	236 (11.3%)		31 (22.1%)	140 (10.2%)		49 (13.0%)	376 (10.9%)	
Upper primary	50 (6.9%)	721 (34.6%)		102 (23.6%)	433 (31.5%)		152 (13.2%)	1154 (33.4%)	
Lower secondary	35 (4.0%)	872 (41.8%)		79 (17.0%)	466 (33.9%)		114 (8.5%)	1338 (38.7%)	
Upper secondary	4 (4.9%)	82 (3.9%)		8 (7.5%)	106 (7.7%)		12 (6.4%)	188 (5.4%)	
Tertiary	0 (0%)	90 (4.3%)		9 (5.8%)	155 (11.3%)		9 (3.7%)	245 (7.1%)	
**Occupation**^1^			0.177			0.001			<0.001
Professionals	5 (3.9%)	127 (6.1%)		2 (3.8%)	53 (3.9%)		7 (3.9%)	180 (5.2%)	
Sales and services	49 (7.3%)	673 (32.3%)		28 (17.6%)	159 (11.6%)		77 (9.3%)	832 (24.1%)	
Skilled manual	0 (0%)	3 (0.1%)		37 (15.2%)	244 (17.8%)		37 (15.0%)	247 (7.1%)	
Unskilled manual	25 (5.1%)	495 (23.7%)		29 (14.7%)	197 (14.3%)		54 (7.8%)	692 (20.0%)	
Agriculture	29 (4.9%)	595 (28.5%)		90 (21.8%)	412 (30.0%)		119 (11.8%)	1007 (29.1%)	
Transport	6 (3.1%)	192 (9.2%)		41 (25.2%)	163 (11.9%)		41 (25.2%)	163 (4.7%)	
Other	0 (0%)	0 (0%)		20 (13.7%)	146 (10.6%)		26 (7.7%)	338 (9.8%)	
**Religion**^2^			0.000			<0.001			<0.001
Catholic	64 (7.9%)	809 (38.8%)		122 (22.0%)	555 (40.4%)		186 (13.6%)	1364 (39.4%)	
Protestant	34 (5.2%)	658 (31.6%)		106 (22.8%)	465 (33.8%)		140 (12.5%)	1123 (32.5%)	
Muslim	6 (2.0%)	299 (14.3%)		10 (4.4%)	226 (16.4%)		16 (3.0%)	525 (15.2%)	
Other	10 (3.1%)	319 (15.3%)		9 (7.0%)	128 (9.3%)		19 (4.3%)	447 (12.9%)	
**Marriage/Union**^3^			0.324			<0.001			<0.001
Not in a marriage/union	57 (6.2%)	916 (43.9%)		93 (15.3%)	607 (44.2%)		150 (9.8%)	1523 (44.0%)	
Consensual	48 (5.3%)	898 (43.1%)		52 (12.5%)	417 (30.3%)		100 (7.6%)	1315 (38.0%)	
Religious	6 (3.5%)	173 (8.3%)		15 (12.8%)	117 (8.5%)		21 (7.2%)	290 (8.4%)	
Traditional	3 (3.1%)	98 (4.7%)		87 (37.3%)	233 (17.0%)		90 (27.2%)	331 (9.6%)	
**No of children**			0.604			<0.001			0.380
0 children	5 (3.5%)	143 (6.9%)		43 (10.2%)	422 (30.7%)		48 (8.5%)	565 (16.3%)	
1 child	20 (6.3%)	319 (15.3%)		36 (20.0%)	180 (13.1%)		56 (11.2%)	499 (14.4%)	
2 to 4 children	56 (5.8%)	971 (46.6%)		94 (20.4%)	461 (33.6%)		150 (10.5%)	1432 (41.4%)	
5+ children	33 (5.1%)	652 (31.3%)		74 (23.8%)	311 (22.6%)		107 (11.1%)	963 (27.8%)	
**Total SES index**^4^			0.602			<0.001			<0.001
Lowest	29 (6.7%)	432 (20.7%)		106 (25.4%)	418 (30.4%)		135 (15.9%)	850 (24.6%)	
Middle-lower	33 (5.5%)	601 (28.8%)		51 (15.4%)	332 (24.2%)		84 (9.0%)	933 (27.0%)	
Middle-upper	22 (5.0%)	443 (21.2%)		33 (13.9%)	238 (17.3%)		55 (8.1%)	681 (19.7%)	
Highest	30 (4.9%)	609 (29.2%)		57 (14.8%)	386 (28.1%)		87 (8.7%)	995 (28.8%)	
**Urbanity-specific SES index**^5^			0.584			<0.001			<0.001
Lowest	28 (6.7%)	420 (20.1%)		102 (25.4%)	402 (29.3%)		130 (15.8%)	822 (23.8%)	
Middle-lower	27 (5.5%)	494 (23.7%)		54 (16.5%)	327 (23.8%)		81 (9.9%)	821 (23.7%)	
Middle-upper	31 (5.4%)	569 (27.3%)		39 (14.0%)	278 (20.2%)		70 (8.3%)	847 (24.5%)	
Highest	28 (4.7%)	602 (28.9%)		52 (14.2%)	367 (26.7%)		80 (8.3%)	969 (28.0%)	
**Urbanity**			0.000			<0.001			<0.001
Urban	26 (3.4%)	767 (36.8%)		52 (11.8%)	439 (32.0%)		78 (6.5%)	1206 (34.9%)	
Semi-urban	58 (9.1%)	636 (30.5%)		96 (21.9%)	438 (31.9%)		154 (14.3%)	1074 (31.0%)	
Rural	30 (4.4%)	682 (32.7%)		99 (19.9%)	497 (36.2%)		129 (10.9%)	1179 (34.1%)	
**Region**			0.007			<0.001			<0.001
Wakiso	67 (6.9%)	968 (46.4%)		60 (10.5%)	570 (41.5%)		127 (8.3%)	1538 (44.5%)	
Hoima	47 (4.2%)	1117 (53.6%)		187 (23.3%)	804 (58.5%)		234 (12.2%)	1921 (55.5%)	
**Smoking**			0.000			<0.001			<0.001
Never	100 (4.9%)	2044 (98.0%)		176 (14.3%)	1227 (89.3%)		276 (8.4%)	3271 (94.6%)	
Former	8 (30.8%)	26 (1.2%)		24 (42.1%)	57 (4.1%)		32 (38.6%)	83 (2.4%)	
Current	6 (40.0%)	15 (0.7%)		47 (52.2%)	90 (6.6%)		53 (50.5%)	105 (3.0%)	
**IPV perpetration past year**^6^			0.022			<0.001			0.016
No	55 (4.5%)	1220 (58.5%)		157 (15.5%)	1010 (73.5%)		212 (9.5%)	2230 (64.5%)	
Yes	59 (6.8%)	865 (41.5%)		90 (24.7%)	364 (26.5%)		149 (12.1%)	1229 (35.5%)	
**IPV victimization past year**^6^			0.064			0.002			0.212
No	56 (4.7%)	1198 (57.5%)		166 (16.1%)	1032 (75.1%)		222 (10.0%)	2230 (64.5%)	
Yes	58 (6.5%)	887 (42.5%)		81 (23.7%)	342 (24.9%)		139 (11.3%)	1229 (35.5%)	
**HIV status**^7^			0.063			0.335			0.662
Negative	94 (5.1%)	1834 (88.0%)		231 (17.7%)	1302 (94.8%)		325 (10.4%)	3136 (90.7%)	
Positive	20 (8.0%)	251 (12.0%)		16 (22.2%)	72 (5.2%)		36 (11.1%)	323 (9.3%)	

Hazardous alcohol use defined as AUDIT-C score ≥3 for women and ≥4 for men. Percentages in *AUDIT-C status* columns are by covariate level. Percentages in *Total* columns are by covariate. Covariate levels not totalling 100% in the *Total* columns are due to rounding. p-values are for the null hypothesis that all covariate levels have the same AUDIT-C status, calculated with Pearson’s chi-squared test.

^1^Occupation groups as by the International Labour Organization’s International Standard Classification of Occupations. *Professionals* group includes technical, clerical, and managerial occupations. *Transport* includes motorcycle and truck drivers. *Other* group includes armed forces, students, and otherwise not classified occupations. ^2^*Protestant* includes Church of Uganda. *Other* group is mostly other Christian denominations, such as Pentecostals. ^3^*Consensual* includes cohabiting. ^4^Household socioeconomic status (SES), measured with an asset index calculated for the full sample. ^5^Household SES, separate indices calculated for rural communities and semi-urban+urban ones. ^6^Includes emotional, physical, and sexual intimate partner violence (IPV). ^7^HIV status is lab verified.

### Sample characteristics

Median age in our sample was 31 years (interquartile range 24–42 years), and 60% (2085/3459) were women. Approximately, half of all participants had attended at least secondary educational institutions, and 56% of both women and men lived in a marriage or union. The most common occupational groups among women were sales or service (32%) and agriculture (29%). For men, agriculture was most common (30%), followed by skilled manual professions (18%). HIV prevalence among women (12%) exceeded twice that of men (5%).

### Prevalence of alcohol use and hazardous alcohol use

**Figure 1.**
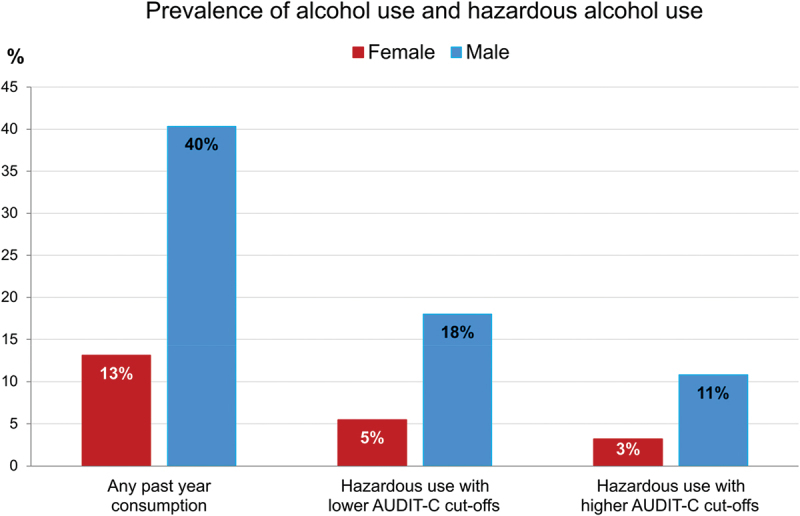
Prevalence of any past-year alcohol use and hazardous use by gender (total *n* = 3459).

Any past-year alcohol consumption was reported at 13% (273/2085) among women and 40% (554/1374) among men (Figure 1).

Data were collected in APHS round 3 from May 2021 to July 2022. Hazardous use defined at lower AUDIT-C cutoffs, ≥3 for women and ≥4 for men, and higher cutoffs, ≥4 for women and ≥5 for men.

Overall, 5% (114/2085) of women and 18% (247/1374) of men screened positive for hazardous alcohol use using AUDIT-C cutoff scores ≥3 for women and ≥4 for men (Table 1). In the total population of 18–20-year-olds, the youngest subset in the sample, 4% (16/390) had a hazardous use of alcohol. The highest age-specific prevalence was 7% (20/285) for 40–49-year-old women and 32% (36/113) for 50–59-year-old men. Among women and men, both sexes with secondary or tertiary education had substantially less hazardous use than those with none or only primary education. Occupational groups with the highest hazardous use were men in the transport sector (25%, 41/163), and female vendors and restaurant or bar workers (7%, 49/673). The largest gender differences per covariate were observed among those living in traditional marriages; 37% of men (87/233) and only 3% of women (3/98) reported hazardous use. The highest prevalence among women, at 9% (58/636), was reported by semi-urban residents. Past-year IPV perpetrators and victims, both male and female, reported higher hazardous use than non-perpetrators or non-victims of IPV. Minimal differences in hazardous use were found between people living with HIV as compared to persons without HIV: 11% (36/323) vs. 10% (325/3136).

With higher cutoff scores, i.e. AUDIT-C ≥ 4 for women and ≥5 for men, 3% of women (66/2085) and 11% of men (148/1374) screened positive for hazardous alcohol use (see Supplementary Table S1 for details).

### Associations between social determinants and hazardous alcohol use

**Figure 2.**
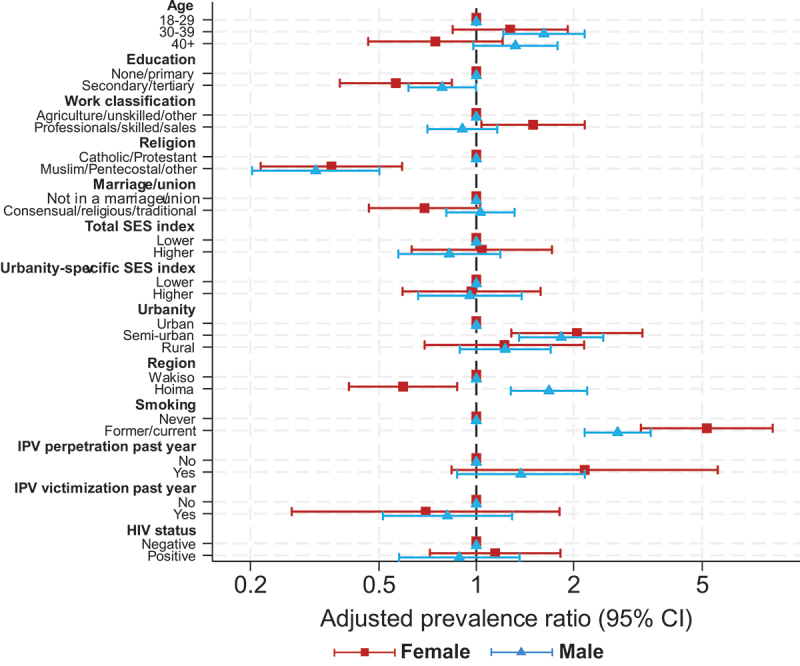
Adjusted prevalence ratios for hazardous alcohol use, separate models per gender (total *n* = 3459).

Crude PRs with 95% CIs are shown in Table 2. Adjusted PRs (aPRs) with 95% CIs are presented in Table 3 and visualized in Figure 2. Among women, hazardous alcohol use was less common among individuals with secondary or tertiary education compared with none or only primary education (aPR 0.6, 95% CI 0.4–0.8), and among those living in a marriage or union (aPR 0.7, 95% CI 0.5–1.0) (Table 3). Skilled and sales or service occupations were associated with higher hazardous use compared with agriculture, unskilled, and other occupations (aPR 1.5, 95% CI 1.0–2.2), as well as semi-urban residency versus urban one (aPR 2.1, 95% CI 1.3–3.3).

Data were collected in round 3 of APHS from May 2021 to July 2022. Hazardous use defined as AUDIT-C score ≥3 for women and ≥4 for men.

Occupational groups followed the ILO’s International Standard Classification of Occupations. *Agriculture/Unskilled/Other*group includes agriculture, unskilled manual, armed forces, students, and otherwise not classified occupations. *Professionals/Skilled/Sales*group includes professional, technical, clerical, managerial, skilled manual, sales, and services occupations. *Protestant* religion includes members of the Church of Uganda, *Other* mostly includes Christian denominations. Household socioeconomic status was measured with an asset index calculated for the full sample. IPV types include emotional, physical, and sexual violence. HIV status is lab verified.

**Table 2.** Crude prevalence ratio for hazardous alcohol use (*n* = 3459).

Variables	Female	Male
	cPR (95% CI)	cPR (95% CI)
**Age**		
18–20	0.68 (0.33–1.38)	0.33 (0.15–0.70)
21–29	1	1
30–39	1.24 (0.80–1.93)	1.52 (1.13–2.04)
40–49	1.28 (0.76–2.16)	1.60 (1.15–2.23)
50+	0.58 (0.29–1.14)	1.68 (1.21–2.33)
**Education**		
None	1.17 (0.56–2.47)	1.05 (0.68–1.61)
Primary	1	1
Lower secondary	0.56 (0.38–0.84)	0.73 (0.57–0.94)
Upper secondary/Tertiary	0.33 (0.12–0.89)	0.28 (0.17–0.46)
**Occupation**^1^		
Skilled	0.79 (0.31–2.00)	0.60 (0.43–0.85)
Sales and services	1.49 (0.96–2.33)	0.81 (0.55–1.18)
Unskilled	1.04 (0.62–1.75)	0.83 (0.62–1.12)
Agriculture	1	1
Other	0.64 (0.27–1.52)	0.79 (0.55–1.13)
**Religion**^2^		
Catholic	1	1
Protestant	0.65 (0.44–0.98)	1.04 (0.82–1.30)
Muslim	0.25 (0.11–0.58)	0.20 (0.11–0.38)
Other	0.40 (0.21–0.76)	0.32 (0.17–0.61)
**Marriage/Union**^3^		
Not in a marriage/union	1	1
Consensual	0.86 (0.59–1.25)	0.81 (0.59–1.12)
Religious	0.56 (0.24–1.27)	0.84 (0.50–1.39)
Traditional	0.49 (0.16–1.54)	2.44 (1.90–3.13)
**No of children**		
0 children	1	1
1 child	1.79 (0.69–4.68)	1.96 (1.31–2.95)
2 to 4 children	1.65 (0.67–4.05)	2.00 (1.43–2.80)
5+ children	1.45 (0.58–3.64)	2.34 (1.65–3.30)
**Total SES index**^4^		
Lowest	1	1
Middle-lower	0.82 (0.50–1.33)	0.61 (0.45–0.82)
Middle-upper	0.74 (0.43–1.27)	0.55 (0.38–0.78)
Highest	0.73 (0.45–1.20)	0.58 (0.44–0.78)
**Urbanity-specific SES index**^5^		
Lowest	1	1
Middle-lower	0.82 (0.49–1.37)	0.65 (0.48–0.87)
Middle-upper	0.82 (0.50–1.34)	0.55 (0.40–0.77)
Highest	0.70 (0.42–1.16)	0.56 (0.41–0.76)
**Urbanity**		
Urban	1	1
Semi-urban	2.69 (1.71–4.22)	1.84 (1.36–2.52)
Rural	1.30 (0.78–2.17)	1.68 (1.23–2.29)
**Region**		
Wakiso	1	1
Hoima	0.61 (0.42–0.87)	2.21 (1.69–2.90)
**Smoking**		
Never	1	1
Former	6.29 (3.43–11.55)	2.94 (2.10–4.10)
Current	8.18 (4.27–15.64)	3.64 (2.86–4.63)
**IPV perpetration past year**^6^		
No	1	1
Yes	1.51 (1.06–2.16)	1.59 (1.26–2.00)
**IPV victimization past year**^6^		
No	1	1
Yes	1.40 (0.98–2.00)	1.47 (1.16–1.86)
**HIV status**^7^		
Negative	1	1
Positive	1.55 (0.98–2.47)	1.25 (0.80–1.96)

Each covariate-gender combination is a separate model.

^1^Occupation groups as by the International Labour Organization’s International Standard Classification of Occupations. *Skilled* group includes professional, technical, clerical, managerial, and skilled manual occupations. *Unskilled* group includes transport. *Other* group includes armed forces, students, and otherwise not classified occupations. ^2^*Protestant* includes Church of Uganda. *Other* group is mostly other Christian denominations, such as Pentecostals. ^3^*Consensual* includes cohabiting. ^4^Household socioeconomic status (SES), measured with an asset index calculated for the full sample. ^5^Household SES, separate indices calculated for rural communities and semi-urban+urban ones. ^6^Includes emotional, physical, and sexual intimate partner violence (IPV). ^7^HIV status is lab verified.

**Table 3.** Adjusted prevalence ratio for hazardous alcohol use (*n* = 3459).

Variables	Female	Male
	aPR (95% CI)	aPR (95% CI)
**Age**		
18–29	1	1
30–39	1.27 (0.84–1.92)	1.62 (1.21–2.16)
40+	0.75 (0.46–1.21)	1.32 (0.98–1.78)
**Education**		
None/Primary	1	1
Secondary/Tertiary	0.56 (0.38–0.84)	0.78 (0.62–1.00)
**Occupation**^1^		
Agriculture/Unskilled/Other	1	1
Professionals/Skilled/Sales	1.50 (1.04–2.16)	0.91 (0.71–1.16)
**Religion**^2^		
Catholic/Protestant	1	1
Muslim/Pentecostal/Other	0.36 (0.22–0.59)	0.32 (0.20–0.50)
**Marriage/Union**^3^		
Not in a marriage/union	1	1
Consensual/Religious/traditional	0.69 (0.47–1.03)	1.03 (0.81–1.31)
**Total SES index**^4^		
Lower	1	1
Higher	1.04 (0.63–1.71)	0.83 (0.57–1.19)
**Urbanity-specific SES index**^5^		
Lower	1	1
Higher	0.97 (0.59–1.58)	0.96 (0.66–1.38)
**Urbanity**		
Urban	1	1
Semi-urban	2.05 (1.28–3.26)	1.83 (1.36–2.47)
Rural	1.22 (0.69–2.16)	1.23 (0.89–1.70)
**Region**		
Wakiso	1	1
Hoima	0.59 (0.40–0.87)	1.68 (1.28–2.20)
**Smoking**		
Never	1	1
Former/Current	5.16 (3.23–8.26)	2.74 (2.16–3.46)
**IPV perpetration past year**^6^		
No	1	1
Yes	2.16 (0.84–5.59)	1.37 (0.87–2.17)
**IPV victimization past year**^6^		
No	1	1
Yes	0.70 (0.27–1.81)	0.81 (0.51–1.29)
**HIV status**^7^		
Negative	1	1
Positive	1.14 (0.72–1.82)	0.89 (0.58–1.36)

Full models, stratified by gender.

^1^Occupation groups as by the International Labour Organization’s International Standard Classification of Occupations. *Agriculture/Unskilled/Other* group includes agriculture, unskilled manual, armed forces, students, and otherwise not classified occupations. *Professionals/Skilled/Sales* group includes professional, technical, clerical, managerial, skilled manual, sales, and services occupations. ^2^*Protestant* includes Church of Uganda. *Other* group is mostly other Christian denominations. ^3^Household socioeconomic status (SES), measured with an asset index calculated for the full sample. ^4^Household SES, separate indices calculated for rural communities and semi-urban+urban ones ^5^*Consensual* includes cohabiting. ^6^Includes emotional, physical, and sexual intimate partner violence (IPV). ^7^HIV status is lab verified.

Among men, secondary or tertiary education compared with none or only primary education (aPR 0.8, 95% CI 0.6–1.0) was associated with lower hazardous use. Higher hazardous use was associated with being in the age range of 30–39 years versus the age range of 18–29 years (aPR 1.6, 95% CI 1.2–2.2) as well as semi-urban residency compared to urban residency (aPR 1.8, 95% CI 1.4–2.5).

Affiliation with Muslim or Pentecostal religion was strongly negatively, and former or current smoking strongly positively correlated with hazardous alcohol use among both women and men when compared to Catholic or Protestant religions (female aPR 0.4 [95% CI 0.2–0.6], male aPR 0.3 [0.2–0.5]), and never having smoked, respectively.

Results indicated a strong positive association between hazardous use and past-year IPV perpetration, for both women (aPR 2.2, 95% CI 0.8–5.6) and men (aPR 1.4, 95% CI 0.9–2.2), and potentially a negative association for IPV victimization: aPR 0.7 for women (95% CI 0.3–1.8) and 0.8 for men (95% CI 0.4–1.3). HIV status was not substantially associated with hazardous alcohol use in the adjusted models.

### Sample representativeness

Among census participants ≥18 years, 52% were female with a median age of 28 years [IQR 23–38], while the corresponding numbers for the survey were 60% female with a median age of 31 years [IQR 24–42]. Average household size in the census was 3.6 persons. At least one household member from 26% of censused households (2321/8847) participated in the survey. Differences in the distribution of participants per study site ranged within five percentage points between the census (11–23%) and the survey (13–21%). The sample included 34% rural, 31% semi-urban, and 35% urban participants. In the 2024 Uganda national census, the average household size was 4.4, among which 52% were female and the median age group was 30–34 years in the population ≥18 years of age [38].

## Discussion

This study in a Ugandan population cohort sought to examine patterns of hazardous alcohol use across a range of social determinants of health. Most participants reported that they had not consumed alcohol in the past year. Hazardous alcohol use was highly gendered, with a prevalence among men three times greater than the prevalence among women (18% vs. 5%).

Hazardous alcohol use was lowest among young adults and increased with age up to 60 years, especially among men. Above age 60, hazardous use decreased. As described in the introduction, men tend to be at a higher risk of hazardous alcohol consumption across the world. Gender discrepancies observed in this study are consistent with prior research in neighbouring Kenya, where estimates for population prevalence of AUD range from 12.3% to 18.1% among men and from 1.3% to 2.2% among women [39,40].

Consistent with extant literature from elsewhere in Africa and beyond, hazardous alcohol use was associated with lower educational attainment [16]. This study found that women working as shop owners and bar or restaurant workers (many with limited schooling) reported higher rates of hazardous alcohol use. Few prior studies have compared hazardous alcohol use among occupation groups in sub-Saharan Africa [8,41,42]. Studies in Tanzania [43] and Uganda [22] found alcohol use to be most prevalent among female bar workers due to the availability and acceptability of drinking in this context, which the results of this study seem to confirm.

No clear association between socioeconomic status and hazardous alcohol use was observed in this study, adding to mixed findings from other African countries on the role of socioeconomic factors [44]. However, in a study in rural Ethiopia, high levels of self-perceived wealth were associated with hazardous alcohol use [45]. Similarly, young men with higher disposable income and a higher number of sexual partners in Northern Tanzania were the most likely to screen positive for AUD [46]. Thus, a more direct measure of individual disposable income could be more informative than measuring household assets accumulated over a longer period.

After controlling for other factors, this study found that both women and men living in semi-urban settings had substantially more hazardous alcohol use than those living in rural or urban areas. Few studies have explored urbanity differences in hazardous alcohol use outside of upper-middle and high-income countries, and existing studies often use a crude rural–urban dichotomization instead of exploring a more realistic rural-to-urban continuum [47]. Given the rapid urbanization of Uganda and many other African countries, both the size and number of peri- or semi-urban communities are increasing. A large proportion of Africa‘s population already lives in such settings, which often are characterized by informal housing and low provision of public services. Thus, their residents may be exposed to unique factors that increase the risk of hazardous use not encountered in deeply rural or highly urban areas [48,49]. Further research should explore whether and how these settings present specific risks.

Religion emerged as a major determinant of hazardous use, likely explained by strong prescriptions on limiting or abstaining from alcohol among Muslim and in some Christian denominations. Smoking was highly predictive of hazardous use, corroborating the well-established overlap between different types of substance use.

In both sexes, hazardous alcohol use was correlated with past-year IPV perpetration. Male partner alcohol use has previously been found to strongly increase the risk of IPV against women [50,51], and the estimated prevalence of IPV against women in East Africa is one of the highest globally [52]. Scant evidence exists on female partner alcohol use and IPV against men [53], despite evidence that male and female IPV perpetrators may share the same risk markers [54] and that alcohol use increases violent behaviour of both men and women [55].

In contrast to the broader evidence base, but consistent with recent work in Uganda by the authors, no clear association was found between hazardous alcohol use and HIV status [22]. Links between alcohol use and increased risk for HIV infection in Uganda which have been previously described include older men buying alcohol for adolescent girls or young women in exchange for (unprotected) sex [56,57].

Factors beyond those explored in our study, such as local social norms, seem to strongly influence hazardous alcohol use and could potentially explain the substantial gender differences found in both this study and previous studies in other African countries. Prior research in rural Uganda noted a pro-alcohol environment in which men overestimated the actual consumption of their peers [58], and drinking was seen as integral to many social activities [59,60]. The alcohol industry in Uganda and other sub-Saharan African countries invests heavily in eye-catching marketing as well as political lobbying aimed at influencing norms around alcohol [61,62]. According to systematic reviews, repeated exposure to alcohol advertising significantly impacts young people‘s perceptions and drinking behaviours [63,64], and alcohol marketing exposure was shown to predict problematic drinking among youth living in the slums of Kampala [65]. In contrast, in European countries, a gender convergence of alcohol consumption patterns has been noted in recent decades [66,67].

Social determinants of health are shaped by political decisions and at the time of writing, a legal debate in Uganda is considering raising the legal drinking age from 18 to 21 [68,69]. These study results show low hazardous use among 18–20-year-olds. Delaying the onset of alcohol use could potentially further reduce hazardous use among young adults and prevent alcohol-related harm in future generations [70,71]. Furthermore, policy measures could target bar and restaurant workers and shop owners to reduce their hazardous use, which according to these findings, would be especially relevant for women. Potential clinical interventions for people with hazardous use include the innovative and locally developed mobile one-week inpatient *Treatment Camp* [72]. In neighbouring Tanzania, the WHO-developed Mental Health Gap Action Programme (mhGAP) module on AUD has been implemented in primary care [73].

### Limitations and strengths

Alcohol consumption in this study was self-reported, carrying the possibility of recall and social desirability bias. The impact of social desirability may have varying gendered effects, with the risk of women underreporting their intake and men inflating their consumption [74]. Nonetheless, previous research in Uganda and neighbouring Tanzania suggests that self-reported measures (via AUDIT) closely align with the alcohol biomarker phosphatidylethanol (PEth), lending credence to these findings [28,75]. Additionally, even though we did not back-translate the questionnaires in Luganda and Runyoro into English and the Runyoro AUDIT version remains to be clinically validated, the authors used established translation and interviewer training procedures to reduce misinterpretations.

The APHS study sites were specifically selected to capture a broad cross-section of Uganda‘s population, covering rural, semi-urban, and urban communities. However, they do not include remote, arid areas near the Sudan and Kenya borders, which are less representative of broader Ugandan society due to their sparse population. Another point limiting the representativeness among men is the lower proportion of men who took part in the survey, relative to the number of men in the household census. Efforts to accommodate men‘s lower likelihood of engaging with surveys were made, as detailed in the cohort description [25] but could not fully mitigate this effect.

Socioeconomic status measures were calculated at the household rather than individual level, a standard method used by large epidemiological surveys. However, such asset indices may not reflect the individual participant‘s actual socioeconomic status. Data on local social norms were not collected and, therefore, their association with hazardous alcohol use could not be assessed. Sub-group analyses were limited by sample size, with the analytic strategy chosen to balance analytical rigor and interpretability of findings.

Overall, this study presents substantial strengths and is one of the largest investigations to date on social determinants of hazardous alcohol use in an African country. The research encompassed diverse Ugandan settings, covered both women and men, and used a comprehensive set of variables selected according to the social determinants of health framework. These design elements and the analytical approach enhance the applicability of the findings to the broader population.

## Conclusions

In a large Ugandan population sample, 5% of women and 18% of men reported hazardous alcohol use. Strongest positive associations with hazardous use were found for individuals living in semi-urban areas, smoking, and past-year perpetration of IPV. Among men, additional factors identified were being middle aged, and among women, additionally being a bar and restaurant or shop workers. Strongest negative associations were found for high educational attainment, Muslim and Pentecostal religion, and for women: living in a marriage or a union. Ultimately, residence in semi-urban areas may present specific risks not encountered in deeply rural or highly urban areas, which warrants further investigation.

## Supplementary Material

Supplemental Material

## Data Availability

The data that support the findings of this study are available from AMBSO. Restrictions apply to the availability of the data, which were used under license for the current study, and are not publicly available. However, data are available from the authors upon reasonable request and with the permission of AMBSO.
