# Supplementary material for: Social determinants of hazardous alcohol use in a Ugandan population cohort
Source: Glob Health Action. 2025 Apr 10;18(1):2484870. doi: 10.1080/16549716.2025.2484870 (PMC11986868; doi:10.1080/16549716.2025.2484870)
Supplement: Supplemental Material [file ZGHA_A_2484870_SM7631.docx]

| **Supplemental Table 1. Descriptive statistics of the study sample (n = 3459), by gender and hazardous alcohol use.** | | | | | | | | | |
| --- | --- | --- | --- | --- | --- | --- | --- | --- | --- |
|  | **WOMEN** | | | **MEN** | | | **TOTAL** | | |
| **VARIABLES** | AUDIT-C ≥4  (n = 66) | Total  (n = 2085) | p-value | AUDIT-C ≥5  (n = 148) | Total  (n = 1374) | p-value | AUDIT-C ≥4 F/≥5 M  (n = 214) | Total (n = 3459) | p-value |
| **Age** |  |  | 0.218 |  |  | <0.001 |  |  | <0.001 |
| 18-20 | 6 (2.5%) | 242 (11.6%) |  | 4 (2.7%) | 148 (10.8%) |  | 10 (2.6%) | 390 (11.3%) |  |
| 21-29 | 22 (3.1%) | 712 (34.1%) |  | 42 (8.7%) | 484 (35.2%) |  | 64 (5.4%) | 1196 (34.6%) |  |
| 30-39 | 21 (4.0%) | 529 (25.4%) |  | 46 (13.6%) | 337 (24.5%) |  | 67 (7.7%) | 866 (25.0%) |  |
| 40-49 | 12 (4.2%) | 285 (13.7%) |  | 32 (15.5%) | 207 (15.1%) |  | 44 (8.9%) | 492 (14.2%) |  |
| 50-59 | 5 (2.7%) | 182 (8.7%) |  | 22 (19.5%) | 113 (8.2%) |  | 27 (9.2%) | 295 (8.5%) |  |
| 60+ | 0 (0%) | 135 (6.5%) |  | 2 (2.4%) | 85 (6.2%) |  | 2 (0.9%) | 220 (6.4%) |  |
| **Highest education** |  |  | 0.075 |  |  | 0.018 |  |  | 0.021 |
| None | 4 (4.8%) | 84 (4.0%) |  | 10 (13.5%) | 74 (5.4%) |  | 14 (8.9%) | 158 (4.6%) |  |
| Lower primary | 12 (5.1%) | 236 (11.3%) |  | 17 (12.1%) | 140 (10.2%) |  | 29 (7.7%) | 376 (10.9%) |  |
| Upper primary | 28 (3.9%) | 721 (34.6%) |  | 53 (12.2%) | 433 (31.5%) |  | 81 (7.0%) | 1154 (33.4%) |  |
| Lower secondary | 20 (2.3%) | 872 (41.8%) |  | 56 (12.0%) | 466 (33.9%) |  | 76 (5.7%) | 1338 (38.7%) |  |
| Upper secondary | 2 (2.4%) | 82 (3.9%) |  | 7 (6.6%) | 106 (7.7%) |  | 9 (4.8%) | 188 (5.4%) |  |
| Tertiary | 0 (0%) | 90 (4.3%) |  | 5 (3.2%) | 155 (11.3%) |  | 5 (2.0%) | 245 (7.1%) |  |
| **Occupation^1^** |  |  | 0.036 |  |  | 0.059 |  |  | <0.001 |
| Professionals | 3 (2.4%) | 127 (6.1%) |  | 0 (0%) | 53 (3.9%) |  | 3 (1.7%) | 180 (5.2%) |  |
| Sales and services | 34 (5.1%) | 673 (32.3%) |  | 17 (10.7%) | 159 (11.6%) |  | 51 (6.1%) | 832 (24.1%) |  |
| Skilled manual | 0 (0.0%) | 3 (0.1%) |  | 23 (9.4%) | 244 (17.8%) |  | 23 (9.3%) | 247 (7.1%) |  |
| Unskilled manual | 13 (2.6%) | 495 (23.7%) |  | 17 (8.6%) | 197 (14.3%) |  | 30 (4.3%) | 692 (20.0%) |  |
| Agriculture | 12 (2.0%) | 595 (28.5%) |  | 50 (12.1%) | 412 (30.0%) |  | 62 (6.2%) | 1007 (29.1%) |  |
| Transport | 4 (2.1%) | 192 (9.2%) |  | 25 (15.3%) | 163 (11.9%) |  | 25 (15.3%) | 163 (4.7%) |  |
| Other | 0 (0%) | 0 (0%) |  | 16 (11.0%) | 146 (10.6%) |  | 20 (5.9%) | 338 (9.8%) |  |
| **Religion^2^** |  |  | 0.033 |  |  | <0.001 |  |  | <0.001 |
| Catholic | 36 (4.4%) | 809 (38.8%) |  | 76 (13.7%) | 555 (40.4%) |  | 112 (8.2%) | 1364 (39.4%) |  |
| Protestant | 19 (2.9%) | 658 (31.6%) |  | 63 (13.5%) | 465 (33.8%) |  | 82 (7.3%) | 1123 (32.5%) |  |
| Muslim | 4 (1.3%) | 299 (14.3%) |  | 6 (2.7%) | 226 (16.4%) |  | 10 (1.9%) | 525 (15.2%) |  |
| Other | 7 (2.2%) | 319 (15.3%) |  | 3 (2.3%) | 128 (9.3%) |  | 10 (2.2%) | 447 (12.9%) |  |
| **Marriage/Union^3^** |  |  | 0.099 |  |  | <0.001 |  |  | <0.001 |
| Not in a marriage/union | 38 (4.1%) | 916 (43.9%) |  | 52 (8.6%) | 607 (44.2%) |  | 90 (5.9%) | 1523 (44.0%) |  |
| Consensual | 24 (2.7%) | 898 (43.1%) |  | 27 (6.5%) | 417 (30.3%) |  | 51 (3.9%) | 1315 (38.0%) |  |
| Religious | 2 (1.2%) | 173 (8.3%) |  | 5 (4.3%) | 117 (8.5%) |  | 7 (2.4%) | 290 (8.4%) |  |
| Traditional | 2 (2.0%) | 98 (4.7%) |  | 64 (27.5%) | 233 (17.0%) |  | 66 (19.9%) | 331 (9.6%) |  |
| **No of children** |  |  | 0.925 |  |  | <0.001 |  |  | 0.368 |
| 0 children | 4 (2.8%) | 143 (6.9%) |  | 22 (5.2%) | 422 (30.7%) |  | 26 (4.6%) | 565 (16.3%) |  |
| 1 child | 12 (3.8%) | 319 (15.3%) |  | 22 (12.2%) | 180 (13.1%) |  | 34 (6.8%) | 499 (14.4%) |  |
| 2 to 4 children | 30 (3.1%) | 971 (46.6%) |  | 60 (13.0%) | 461 (33.6%) |  | 90 (6.3%) | 1432 (41.4%) |  |
| 5+ children | 20 (3.1%) | 652 (31.3%) |  | 44 (14.1%) | 311 (22.6%) |  | 64 (6.6%) | 963 (27.8%) |  |
| **Total SES status^4^** |  |  | 0.704 |  |  | 0.028 |  |  | <0.001 |
| Lowest | 17 (3.9%) | 432 (20.7%) |  | 64 (15.3%) | 418 (30.4%) |  | 81 (9.5%) | 850 (24.6%) |  |
| Middle-low | 17 (2.8%) | 601 (28.8%) |  | 34 (10.2%) | 332 (24.2%) |  | 51 (5.5%) | 933 (27.0%) |  |
| Middle-high | 15 (3.4%) | 443 (21.2%) |  | 17 (7.1%) | 238 (17.3%) |  | 32 (4.7%) | 681 (19.7%) |  |
| Highest | 17 (2.8%) | 609 (29.2%) |  | 33 (8.5%) | 386 (28.1%) |  | 50 (5.0%) | 995 (28.8%) |  |
| **Urbanicity-specific SES status^5^** |  |  | 0.270 |  |  | 0.002 |  |  | 0.001 |
| Lowest | 16 (3.8%) | 420 (20.1%) |  | 58 (14.4%) | 402 (29.3%) |  | 74 (9.0%) | 822 (23.8%) |  |
| Middle-low | 11 (2.2%) | 494 (23.7%) |  | 35 (10.7%) | 327 (23.8%) |  | 46 (5.6%) | 821 (23.7%) |  |
| Middle-high | 23 (4.0%) | 569 (27.3%) |  | 25 (9.0%) | 278 (20.2%) |  | 48 (5.7%) | 847 (24.5%) |  |
| Highest | 16 (2.7%) | 602 (28.9%) |  | 30 (8.2%) | 367 (26.7%) |  | 46 (4.7%) | 969 (28.0%) |  |
| **Urbanicity** |  |  | 0.002 |  |  | 0.002 |  |  | <0.001 |
| Urban | 19 (2.5%) | 767 (36.8%) |  | 32 (7.3%) | 439 (32.0%) |  | 51 (4.2%) | 1206 (34.9%) |  |
| Semi-urban | 33 (5.2%) | 636 (30.5%) |  | 64 (14.6%) | 438 (31.9%) |  | 97 (9.0%) | 1074 (31.0%) |  |
| Rural | 14 (2.1%) | 682 (32.7%) |  | 52 (10.5%) | 497 (36.2%) |  | 66 (5.6%) | 1179 (34.1%) |  |
| **Region** |  |  | 0.928 |  |  | <0.001 |  |  | 0.001 |
| Wakiso | 31 (3.2%) | 968 (46.4%) |  | 40 (7.0%) | 570 (41.5%) |  | 71 (4.6%) | 1538 (44.5%) |  |
| Hoima | 35 (3.1%) | 1117 (53.6%) |  | 108 (13.4%) | 804 (58.5%) |  | 143 (7.4%) | 1921 (55.5%) |  |
| **Smoking** |  |  | <0.001 |  |  | <0.001 |  |  | <0.001 |
| Never | 58 (2.8%) | 2044 (98.0%) |  | 98 (8.0%) | 1227 (89.3%) |  | 156 (4.8%) | 3271 (94.6%) |  |
| Former | 5 (19.2%) | 26 (1.2%) |  | 19 (33.3%) | 57 (4.1%) |  | 24 (28.9%) | 83 (2.4%) |  |
| Current | 3 (20.0%) | 15 (0.7%) |  | 31 (34.4%) | 90 (6.6%) |  | 34 (32.4%) | 105 (3.0%) |  |
| **IPV perpetration past year^6^** |  |  | 0.093 |  |  | <0.001 |  |  | 0.003 |
| No | 32 (2.6%) | 1220 (58.5%) |  | 86 (8.5%) | 1010 (73.5%) |  | 118 (5.3%) | 2230 (64.5%) |  |
| Yes | 34 (3.9%) | 865 (41.5%) |  | 62 (17.0%) | 364 (26.5%) |  | 96 (7.8%) | 1229 (35.5%) |  |
| **IPV victimization past year^6^** |  |  | 0.460 |  |  | 0.001 |  |  | 0.240 |
| No | 35 (2.9%) | 1198 (57.5%) |  | 95 (9.2%) | 1032 (75.1%) |  | 130 (5.8%) | 2230 (64.5%) |  |
| Yes | 31 (3.5%) | 887 (42.5%) |  | 53 (15.5%) | 342 (24.9%) |  | 84 (6.8%) | 1229 (35.5%) |  |
| **HIV status^7^** |  |  | 0.430 |  |  | 0.493 |  |  | 0.334 |
| Negative | 56 (3.1%) | 1834 (88.0%) |  | 142 (10.9%) | 1302 (94.8%) |  | 198 (6.3%) | 3136 (90.7%) |  |
| Positive | 10 (4.0%) | 251 (12.0%) |  | 6 (8.3%) | 72 (5.2%) |  | 16 (5.0%) | 323 (9.3%) |  |

Hazardous alcohol use defined as AUDIT-C score ≥4 for women and ≥5 for men.
Percentages in *AUDIT-C status* columns are by covariate level. Percentages in *Total* columns are by covariate. Covariate levels not totalling 100% in the *Total* columns are due to rounding. p-values are for the null hypothesis that all covariate levels have the same AUDIT-C status, calculated with Pearson's chi-squared test.
^1^ Occupation groups as by the International Labour Organization’s International Standard Classification of Occupations. *Professionals* group includes technical, clerical, and managerial occupations. *Transport* includes motorcycle and truck drivers. *Other* group includes armed forces, students, and otherwise not classified occupations. ^2^ *Protestant* includes Church of Uganda. *Other* group is mostly other Christian denominations, such as Pentecostals. ^3^ *Consensual* includes cohabiting. ^4^ Household socioeconomic status (SES), measured with an asset index calculated for the full sample. ^5^ Household SES, separate indices calculated for rural communities and semi-urban+urban ones. ^6^ Includes emotional, physical and sexual intimate partner violence (IPV). ^7^ HIV status is lab verified.
